# Factors influencing weight control practices amongst the adolescent girls in Vhembe District of Limpopo Province, South Africa

**DOI:** 10.4102/phcfm.v8i2.952

**Published:** 2016-05-31

**Authors:** Rose Tshililo, Lizzy M. Netshikweta, Grace T. Tshitangano, Hilda L. Nemathaga

**Affiliations:** 1 Department of Advanced Nursing Science, University of Venda, South Africa; 2 Department Public Health, University of Venda, South Africa

## Abstract

**Background:**

The incidence of overweight is increasing amongst adolescents in many countries around the world. Healthy and unhealthy weight control practices are common amongst overweight and non-overweight adolescents.

**Aim:**

The aim of this study was to explore factors influencing weight control practices amongst adolescent girls.

**Setting:**

The study was conducted at selected secondary schools of Vhembe District of Limpopo Province, South Africa.

**Methods:**

A qualitative, exploratory, descriptive and contextual design was used. Non-probability, purposive sampling was used to select adolescents who are practicing weight control. In-depth interviews were conducted with 30 participants. Data were analysed according to Tesch’s open-coding method.

**Results:**

This study revealed that adolescent girls are influenced by a variety of factors to control their weights. These included individual factors, such as body image dissatisfaction; family factors, caused by parental criticism about adolescent weight; and environmental factors, which contain peer group endorsement of dieting.

**Conclusion:**

Adolescents are exposed to many unhealthy weight control practices, as a way of controlling excess weight. So it is of importance for healthcare providers to make them aware of healthy practices.

## Introduction

There is a global concern about weight control amongst adolescents. Adolescent girls are mostly exposed to the unrealistically thin beauty ideal that is portrayed in the media. Unfortunately, this overemphasis on the importance of being thin is internalised by youth who equate thinness with beauty, success and health. In addition, adolescents are exposed to a number of ways to lose weight and achieve this thin ideal. The result is that many adolescents feel the pressure to be thinner than is required for good health and may try to achieve this goal through poor and sometimes dangerous nutritional choices.^1^

Adolescent girls are known to be interested in losing weight because of their concerns about their body weight. Healthy and unhealthy weight control practices are also common for overweight and non-overweight adolescents. Amongst the unhealthy practices, fasting, skipping meals, and eating very little food were the most common in South Africa. Authors indicated that unhealthy weight control practices include fasting, eating small amounts of food, using a food substitute (powdered substitutes or a special drink), skipping meals and excessive smoking. Extreme weight control practices include using appetite suppressants, induced vomiting, using laxatives and using diuretics.^2^

Studies revealed that unhealthy weight control practices are associated with potential negative physical health consequences; these include physical and psychological consequences. Physical factors include stunting and malnutrition and psychological factors include bulimia and anorexia nervosa. Furthermore, adolescents with lower self-esteem are more likely to diet, often in an attempt to feel better about themselves if weight loss is successful. It was revealed that such practices would probably increase weight gain instead of weight loss. Studies also indicated that skipping breakfast, in particular, could lead to nutritional problems and interfere with learning and academic performance. Skipping meals could also lead to more extreme weight control practices, such as the use of diet pills and self-induced vomiting.^2,3^

### Purpose of the study

The aim of this study was to investigate factors influencing weight control practices amongst adolescent girls.

### Objectives

Explore and describe the factors influencing weight control practices amongst adolescent girls.

## Research methods and design

### Study design

A qualitative, exploratory, descriptive and contextual design was followed using focus group discussions to explore weight control practices amongst adolescents in Vhembe District of Limpopo Province, South Africa.^4^ Qualitative research is defined as the inquiry into phenomena, normally in an in-depth and holistic fashion, through the collection of rich narrative material using a flexible research design.

### Study population and sampling strategy

The target population in this study comprised 50 adolescents who practiced different strategies to control their weight. They were from selected secondary schools in Vhembe district.

Non-probability purposive sampling was used to select adolescents aged 13–19 years who practiced weight control and who were willing to participate in the study. Research was conducted at the schools. The sample size was 1 focus group from 5 participating schools, each focus group comprised 10 participants, making a total of 50 participants after which data saturation was reached.

### Data collection

Five focus group discussions (with 10 participants each) were conducted to collect data regarding factors influencing weight control practices amongst adolescent girls from selected schools in Vhembe district. Permission to conduct the study was obtained from the Department of Education as well as principals from the sampled schools. Consent forms for focus group participants were completed in advance by adolescents willing to participate. Data were audio-recorded and field notes were taken. Observations were made during focus groups. The recorded interviews were transcribed verbatim.^4^

### Data analysis

Qualitative data were analysed using Tesch’s eight steps of open-coding.^4^ Themes and sub-themes emerged and they reflected that adolescent girls are influenced by a variety of factors to control their weights. These include individual factors, family factors and environmental factors.

### Trustworthiness

Credibility was ensured by prolonged engagement, persistent observation and triangulation. A full description of the research methodology was provided to external experts to ensure dependability. For conformability, raw data were audio-recorded during the focus groups with the participants and the researcher provided sufficient descriptive data in the report for consumers to evaluate the applicability of the data to other contexts to ensure transferability.^1^

### Ethical considerations

Approval for the study was obtained from the Univen Venda Higher Degrees Committee as well as the University of Venda Health, Safety and Research Ethics Committee and approval was granted (SHS 4/12/PH/03/0812). Permission was sought from the Provincial Department of Health and Welfare and District Department of Health. Informed consent was obtained from participants after giving full information about the purpose of the study using the language that they understood. Ethical principles of voluntary participation, informed consent and confidentiality were considered throughout the study. The researcher ensured that all collected information was stored and used in such a manner that the confidentiality, anonymity and privacy of all participants were maintained, bearing in mind the sensitivity of the research. The rights, needs, values and desires of the participants were protected. The participants were assured that they had the right to refuse to participate in the study without any penalty and also to withdraw from the study at any time they wished.

## Results

The study findings confirmed that adolescent girls are influenced by a variety of factors to unhealthy weight control practice, including individual, family and environmental factors.

### Participants’ characteristics

There were five focus groups, each with 10 participants and all were girls. The majority of them were between the ages of 15 and 17 years and they were in grade 10 to grade 12 from five sampled schools. The study identified the following themes related to the factors influencing weight control practices (Table 1)

**TABLE 1:** Themes and sub-themes that emerged during data analysis.

Themes	Sub-themes
1. Participant expressed personal feelings of discontent about their appearances	1.1 Body image dissatisfaction 1.2 Overweight and obesity 1.3 Insufficient knowledge about nutrition
2. Adolescents experience family teasing and false teachings about their weight	2.1 Parental criticism 2.2 Parental dieting 2.3 Parental encouragement on diet
3. Peer pressure determines adolescent weight control practice	3.1 Weight-related teasing 3.2 Peer group endorsement of dieting

*Source:* Authors’ own work

### Theme 1: Participant expressed personal feelings of discontent about their appearances

Participants were dissatisfied about their body images. They indicated that beauty is concerned with thinness. Participant responded in this manner:

‘I’m really not satisfied about my image, because of this overweight of mine. When I buy clothes I have to buy those that are meant for adult people and this scared me so what must I do? I told myself to skip meals and I sometimes eat one apple the whole day.’ (Female 17, grade 12)

‘I eat less to lose weight and if I realised that I ate too much, I put my fingers in the mouth to induce vomiting because I am not satisfied with my weight. Others even called me ‘bumba’ and I don’t like those nick-names.’ (Female 15, grade 10)

Participants also showed insufficient knowledge regarding healthy weight control strategies. Others indicated that by practicing the unhealthy practices they successfully lost weight as they were scared about obesity and overweight. One participant expressed herself in this way:

‘Before I started taking water with lemon the whole day, I was weighing 90 kg. Since I started practising that, I lost 20 kg, so I considered taking water with lemon the whole day as a good strategy to lose weight.’ (Female 17, grade 12)

### Theme 2: Adolescents experience family teasing and false teachings about their weight

Participants indicated that they are often influenced by their relatives to practice unhealthy ways to control weight and this is by parental criticism about the adolescent’s weight, parental dieting and parental encouragement to diet:

‘My mother is a very slender person with a beautiful figure; she does not always and also encourage us not to eat, so I am following her as I skip most of the meals, I usually eat in the evening.’ (Female 16, grade 11)

‘I am the only one with overweight in my family and everyone is commenting negatively on me and I bought slimming tablets to reduce my weight and I don’t eat during the day.’ (Female 17, grade 12)

Wang et al.^5^ argued that specific parenting strategies related to meals and physical activity are protective against unhealthy weight control practices amongst the adolescents. The importance of family meals and strategies to facilitate family meals should be incorporated into discussions between healthcare providers, parents, and children to promote healthy weight-related practices.

### Theme 3: Peer pressure determines adolescent weight control practice

This study revealed that adolescents are influenced by environmental factors to unhealthy weight control practices. These include home, school, physical and social environment. McAdams^6^ considered home environment as the most influential setting for shaping children’s eating and physical activity behaviours:

‘I took the advice from my friends at school that I can lose weight by skipping meals and taking slimming. I accepted the advice as they all have good figures.’ (Female 17, grade 12)

‘My school mates are not eating during school brake, instead they take snacks. They practice this as a way of maintaining their weight; I joined them as I also want to control my weight. Before I joined them, they were teasing me about my weight.’ (Female 16, grade 11)

## Discussion

Weight-related problems, including obesity, inadequate physical activity, poor eating behaviours, unhealthy weight control practices and body dissatisfaction, are prevalent amongst adolescent girls. This study revealed that adolescent girls are dissatisfied about their body image, and as a result, they practise many unhealthy practices to control their weights as they also link thinness with beauty. Despite the negative impact of obesity and overweight, healthy weight control practices need to be adhered to. Participants are dissatisfied about obesity and overweight; however, they lack the necessary knowledge on how to control their weight. Many adolescents globally are overweight, and if they consider themselves as overweight, they are more likely to practice dieting.^2,3,7^

This also supports the study findings that adolescents practise unhealthy eating behaviours, which includes excessive consumption of water and snacking at nighttime.^3,8^ The participants indicated that they practice self-induced vomiting and eating less in order to lose weight. Cynthia and McAdams^6^ claimed that self-induced vomiting is associated with sedentary behaviours and unhealthy eating habits, which are the same risk factors for developing obesity later in life.^6^ Adolescents lack information of healthy weight control practices; instead, they obtain unreliable information from the Internet and other unreliable sources. As a result, they are likely to use unhealthy weight control strategies.^9^

The study revealed that most of the factors that influence adolescents to practice unhealthy weight control practices are imposed by family members. This means that parents also lack the necessary knowledge to guide their children on the importance of a well-balanced diet and physical activity. Neumark-Sztainer et al.^10^ argued that new moves designed to prevent a broad range of weight-related problems in adolescent girls impacted positively in controlling unhealthy weight control practices. Weight-related talk and weight-related teasing by family members resulted in problematic weight-related outcomes and greater body dissatisfaction amongst adolescent girls and they are more likely to engage in unhealthy and extreme weight control practices.

This study also revealed that adolescents are influenced by their peers. Peer pressure is the most determinant factor for adolescence lifestyle; they often consider them as their role models. Liou^3^ considered the school environment as an appropriate setting for the promotion of health interventions amongst school children. School curriculum should be combined with health education that includes information about healthy dieting and physical activity.

### Recommendations

The following recommendations were made:

The Department of Health through school health nursing should educate adolescents about healthy strategies to control their weight. The school curriculum should include health education topics, such as healthy eating and physical activity as a way of controlling and maintaining weight. Roles of parents and family members regarding adolescents’ education on healthy eating and physical activity to control weight should be well communicated. Further research about healthy eating and physical activity should be done.

## Conclusion

Adolescents are exposed to many unhealthy weight control practices, as a way of controlling excess weight. It is of importance for healthcare providers to make adolescents aware of healthy weight loss practices. Health education should include information on ways to lose diet in a healthy way that will not compromise the adolescent’s health.
